# Bringing Molecules Back into Molecular Evolution

**DOI:** 10.1371/journal.pcbi.1002572

**Published:** 2012-06-28

**Authors:** Claus O. Wilke

**Affiliations:** Section of Integrative Biology, Center for Computational Biology and Bioinformatics, and Institute of Cell and Molecular Biology, The University of Texas at Austin, Austin, Texas, United States of America; University of California San Diego, United States of America

## Abstract

Much molecular-evolution research is concerned with sequence analysis. Yet these sequences represent real, three-dimensional molecules with complex structure and function. Here I highlight a growing trend in the field to incorporate molecular structure and function into computational molecular-evolution work. I consider three focus areas: reconstruction and analysis of past evolutionary events, such as phylogenetic inference or methods to infer selection pressures; development of toy models and simulations to identify fundamental principles of molecular evolution; and atom-level, highly realistic computational modeling of molecular structure and function aimed at making predictions about possible future evolutionary events.

This is an “Editors' Outlook” article for *PLoS Computational Biology*.

## Introduction

The field of molecular evolution investigates how genes and genomes evolve over time. It has its origin in the late 1960s, when the first DNA and protein sequences were becoming available. With rapid progress in sequencing technologies came ever increasing demand for computational tools to study molecular evolution. Today, molecular evolution is among the largest subfields of evolutionary biology, and arguably one of the most computationally advanced. Thousands of person years have gone into developing sophisticated alignment algorithms, phylogenetic-tree reconstruction methods, or statistical tests for positive selection.

A side effect of the strong emphasis on developing sophisticated methods for sequence analysis has been that the underlying biophysical objects represented by the sequences, DNA molecules, RNA molecules, and proteins, have taken a back-seat in much computational molecular-evolution work. The vast majority of algorithms for sequence analysis, for example, incorporate no knowledge of biology or biochemistry besides that DNA and RNA sequences use an alphabet of four letters, protein sequences use an alphabet of 20, and the genetic code converts one into the other. The choice to treat DNA, RNA, and proteins simply as strings of letters was certainly reasonable in the late 20th century. Computational power was limited and many basic aspects of sequence analysis were still relatively poorly understood. However, in 2012 we have extremely powerful computers and a large array of highly sophisticated algorithms that can analyze strings of letters. It is now time to bring the molecules back into molecular evolution. Several groups have embarked on this path, and I will highlight some of the work that has been done and speculate on future developments we may see.

In this article, I focus on the evolution of protein-coding genes, the area I am most familiar with myself. However, my overall message, that it is time to bring the molecules back into molecular evolution, similarly applies to other genetic sequences, such as intergenic regions, RNA genes, or the various forms of short RNAs. I will consider three broad areas, corresponding to three distinct research goals: (i) reconstructing and interpreting past evolutionary events; (ii) identifying fundamental principles of molecular evolution; and (iii) predicting probable evolutionary trajectories.

## Reconstructing and Interpreting Past Evolutionary Events

A major goal of comparative sequence analysis is to reconstruct and/or interpret past evolutionary events. For example, we may have sequences from multiple species and want to know how they relate to each other, which specific sequence changes caused them to diverge, and whether certain sites were under particularly strong selective pressure. The standard analysis pipeline for such questions is to align sequences, build trees, and run scans for positive or other types of selection, and/or for recombination. This analysis pipeline uses nothing but sequences as input. Only once the analysis is completed may the researchers take sites of interest they have identified, map them back onto the structure of the protein they are studying, and carry out further experimentation. (However, increasingly the initial sequence analysis is only the prerequisite for a successful study, and the value of the study is defined by the follow-up work; see e.g., [1], [2].)

The standard analysis approach has been highly successful. Yet it ignores most of the biochemistry that ultimately determines the fitness landscape in which sequences evolve. Thus, methods that combine sequence data with additional information, such as protein structure, should yield more sensitive and more accurate estimates than methods based on sequence data alone. On the basis of this premise, a few groups have started to develop such methods. For example, some authors have developed models of coding-sequence evolution that incorporate interactions among sites mediated by protein structure [3]–[5]. (See also this review: [6].) Similarly, some authors have incorporated knowledge of protein structure in methods of ancestral state reconstruction [7]. Finally, in phylogenetic-tree inference, evidence is accumulating that independence of sites may not be a good assumption [8] for protein-coding and even more so for RNA-coding sequences. Thus, future methods of phylogenetic tree reconstruction may also incorporate structural information in some form. Coarse-grained models of protein-sequence evolution are being developed that may be useful for this purpose [9].

The development of methods that integrate molecular structure into sequence analysis is still in its infancy. While several groups are exploring a variety of approaches, none of these approaches is well established at this time. Comparative analyses that use nothing but sequence data remain state of the art. My expectation for the near future is that we will continue to see efforts to extend comparative analyses beyond sequence data alone. Eventually, some of these efforts will prove sufficiently useful that it will become commonplace to combine sequence data with structural, functional, or other molecular data in comparative analyses.

## Identifying Fundamental Principles of Molecular Evolution

Besides understanding and interpreting specific evolutionary events, evolutionary biologists also aim to identify fundamental principles of molecular evolution. Fundamental principles are insights that apply to many different biological systems; a classical example would be the finding that codon usage bias correlates with gene expression level [10], [11].

The search for fundamental principles tends to require somewhat different computational approaches than the analysis of past evolutionary events. It often involves developing toy models (either in the form of mathematical equations or of simulations) to explore possible system dynamics under different modeling assumptions or parameter choices. The specific toy models to be explored are usually inspired by observations from past evolutionary events. To give an example from my own research, starting about 10 years ago many groups found that highly expressed proteins evolve slowly [12]. This observation prompted several authors to develop models of varying complexity that might explain the pattern [13]–[17].

Toy models of evolution have been studied for over a century. And much of this work has not considered the underlying biochemistry of the evolving organism. For example, the population-genetics literature contains plenty of abstract, mathematical models (such as two-locus, two-allele models) that make absolutely no assumptions about the mechanisms that connect different allelic states with different fitness values. These abstract mathematical models are valuable, of course, yet they can get us only so far. Most importantly, they cannot explain how, mechanistically, genotype maps to phenotype and fitness.

As we try to get a better understanding of the genotype-phenotype map, we have to build more realistic models. For example, virtually all the models trying to explain the relationship between evolutionary rate and expression level make concrete assumptions about mechanisms of protein folding and function [13]–[17]. Many implement an actual (though simplified) protein-folding model in which actual amino-acid sequences are computationally folded, using either a lattice [14], [15] or an off-lattice [16] approach.

More generally, we are seeing an increased trend towards integrating some biophysical realism into toy models of evolution. Several groups are regularly working with models that incorporate some aspect of protein biochemistry, such as protein fold stability [9], [14]–[16], [18]–[22] or protein–protein interactions [22]–[24]. Models may represent individual evolving proteins [18], [20], [21], [23] or entire cells [19], [22], [24]. Finally, some groups elucidate the molecular fitness landscapes that underlie adaptive events [25]–[27]. Works such as these aim to identify the biophysical mechanisms that drive molecular evolution.

I believe that we have only scratched the surface of what is possible with simple, biophysically inspired models of molecular evolution. I expect that we are going to see more of this modeling approach in the coming years, and that it will help us to develop a deeper understanding of fundamental principles of molecular evolution.

## Predicting Probable Evolutionary Trajectories

For many real-world applications, it would be useful to be able to predict future evolutionary events. For example, we know that H5N1 avian influenza could potentially cause a deadly pandemic if it ever evolved the ability to effectively spread between humans. What we do not know [28] is the likelihood that it will evolve this ability, nor whether it might possibly become less pathogenic as it evolves more effective human-to-human transmission capabilities. As a second example, some authors have proposed treating infectious diseases with interfering particles (e.g., [29]). Because of the potential for transmission of these particles among infected patients, the safety of such treatments stands and falls with our ability to accurately predict how such therapeutic particles might evolve once released.

Since evolution is a stochastic process, we cannot expect to ever predict which specific mutations will accumulate in a given lineage. However, at least in principle, we should be able to make probabilistic predictions of the form “Outcome A is the most likely, and has a 37% probability of occurring; outcome B is the second most likely, and has a 24% probability of occurring.” It would be tremendously useful if we could make such predictions reliably, in particular for rapidly evolving pathogens. Therefore, there is growing interest among evolutionary biologists to develop predictive frameworks [30]–[33]. In my opinion, successful approaches in this area will most likely involve realistic, atom-level computational modeling of the system of interest.

With rapid increases in computational power over the last two decades, realistic modeling of biological systems is becoming increasingly feasible. At the molecular level, obvious applications of realistic modeling are atom-level predictions of protein structure [34] or protein-folding dynamics [35], [36], and computational enzyme design [37]–[39]. The accuracy of these computational models, when they work, is getting quite good. For example, in computational enzyme design, where the goal is to design catalytically active enzymes de novo, crystal structures of successfully designed enzymes are often very close to the computationally predicted ones [39]. However, it is common that only a small number of the computational designs actually work as expected. In a recent study, 84 computationally designed enzymes were evaluated experimentally [39]. Of those, 50 were soluble and only two catalyzed the desired reaction.

At present, atom-level modeling of proteins is not commonly used in applications of evolutionary biology (but see [40]). However, it seems to me that as our modeling capability improves, a logical next step will be to apply these models to predicting evolution. If we can predict computationally which mutants will be able to carry out specific functions, then we should also be able to predict which mutants are likely to arise under specific, well-defined selection pressures. While I cannot imagine that we will ever be able to solve open-ended problems, such as, for example, to predict all the sequence changes an invasive species will undergo as it is introduced into a new environment, we should have reasonable success for well-defined problems, such as to find the mutations an animal virus would require to bind to the human form of the receptor it uses for cell entry in its host species.

An alternative to atom-level modeling can be statistical inference of biophysically important sites from large sequence alignments. For example, in a recent paper Bloom and Glassman [41] proposed a method to infer the effect of point mutations on protein stability from the distribution of mutations in a dense phylogeny. This method performed better in predicting measured 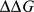 values than alternative methods based on protein structure and atomic force fields. Bloom and co-workers then used this method to identify mutations that were likely to be involved in the evolution of oseltamivir resistance in influenza [42].

Regardless of whether one uses atom-level modeling or statistical approaches, computational predictions are not going to be perfect. Thus, computational methods to predict evolution are most likely going to be useful in generating candidate scenarios. These candidate scenarios will include many false positives and will have to be screened experimentally to separate false from true positives.

## Summary

There is a growing trend in widely differing subfields of molecular evolution to increase biophysical realism in computational models of sequence evolution. Some subfields are further along this path than others. Among groups developing simple toy models of evolution, models incorporating some biophysical realism have been quite popular in recent years. By contrast, statistical models of sequence evolution incorporating biophysical realism are being developed by some groups but are not being routinely applied in sequence-analysis applications. A major impediment to more routine use of such models is likely the lack of widely available, easy-to-use implementations. Hopefully, we will see progress in this area soon. Methods to predict future evolutionary trajectories do not really exist at this time. However, there is a growing interest in developing them. I believe that the computational methods required for this type of prediction are falling into place in the protein-design field; we may soon see a first, small-scale demonstration that computational prediction of evolutionary trajectories is actually possible.

**Author's Biography**

**Claus O. Wilke** is an Associate Professor in the Section of Integrative Biology at The University of Texas at Austin. He is also a member of the Center for Computational Biology and Bioinformatics and the Institute for Cell and Molecular Biology at The University of Texas at Austin. Dr. Wilke received his PhD in theoretical physics from the Ruhr-University Bochum, Germany, in 1999. From 2000 to 2004, he was a postdoc at the California Institute of Technology, working on viral evolution and artificial life. After his postdoc, he spent a year as a Research Assistant Professor at the Keck Graduate Institute of Applied Life Sciences, Claremont, before joining The University of Texas in the fall of 2005. His current research interests are in molecular evolution, structural biology, and biostatistics. Dr. Wilke is the author of approximately 100 scientific papers. He serves as Associate Editor for *PLoS Computational Biology* and *PLoS Pathogens*, and as Section Editor for *BMC Evolutionary Biology*. In 2011, Dr. Wilke was recognized as a Leading Texas Innovator by The Academy of Medicine, Engineering, and Science of Texas.
